# Comparative Transcriptomics Reveals Distinct Gene Expressions of a Model Ciliated Protozoan Feeding on Bacteria-Free Medium, Digestible, and Digestion-Resistant Bacteria

**DOI:** 10.3390/microorganisms8040559

**Published:** 2020-04-13

**Authors:** Songbao Zou, Qianqian Zhang, Jun Gong

**Affiliations:** 1 Yantai Institute of Coastal Zone Research, Chinese Academy of Sciences, Yantai 264003, China; songbaozou@126.com; 2 University of Chinese Academy of Sciences, 19 Yuquan Road, Beijing 100049, China; 3 Center for Ocean Mega-Science, Chinese Academy of Sciences, 7 Nanhai Road, Qingda 266071, China; 4 School of Marine Sciences, Sun Yat-Sen University, Zhuhai Campus, Tangjiawan, Zhuhai 519000, China; 5 Southern Marine Science and Engineering Guangdong Laboratory (Zhuhai), Zhuhai 519000, China

**Keywords:** protozoa, bacterivory, feeding, gene expression, microbial loop, RNA-seq

## Abstract

Bacterivory is an important ecological function of protists in natural ecosystems. However, there are diverse bacterial species resistant to protistan digestion, which reduces the carbon flow to higher trophic levels. So far, a molecular biological view of metabolic processes in heterotrophic protists during predation of bacterial preys of different digestibility is still lacking. In this study, we investigated the growth performance a ciliated protozoan *Tetrahymena* *thermophila* cultivated in a bacteria-free Super Proteose Peptone (SPP) medium (control), and in the media mixed with either a digestion-resistant bacterial species (DRB) or a digestible strain of *E. coli* (ECO). We found the protist population grew fastest in the SPP and slowest in the DRB treatment. Fluorescence in situ hybridization confirmed that there were indeed non-digested, viable bacteria in the ciliate cells fed with DRB, but none in other treatments. Comparative analysis of RNA-seq data showed that, relative to the control, 637 and 511 genes in *T. thermophila* were significantly and differentially expressed in the DRB and ECO treatments, respectively. The protistan expression of lysosomal proteases (especially papain-like cysteine proteinases), GH18 chitinases, and an isocitrate lyase were upregulated in both bacterial treatments. The genes encoding protease, glycosidase and involving glycolysis, TCA and glyoxylate cycles of carbon metabolic processes were higher expressed in the DRB treatment when compared with the ECO. Nevertheless, the genes for glutathione metabolism were more upregulated in the control than those in both bacterial treatments, regardless of the digestibility of the bacteria. The results of this study indicate that not only bacterial food but also digestibility of bacterial taxa modulate multiple metabolic processes in heterotrophic protists, which contribute to a better understanding of protistan bacterivory and bacteria-protists interactions on a molecular basis.

## 1. Introduction

Heterotrophic protists (protozoa) are major consumers of bacteria, controlling the abundance and community composition of bacteria and regenerating nutrients for autotrophs in aquatic and soil ecosystems [1,2]. Effective ingestion and digestion of bacterial preys by protists are essential to fulfill their pivotal role in channeling organic matter and energy to higher trophic levels via the microbial loop. During this process, however, many bacterial species seem to have evolved a range of physiological, behavior, and chemical mechanisms to avoid being ingested (e.g., small or large cell sizes, rapid swimming, surface masking, and microfilm formation) or being intracellularly digested (e.g., toxin release, intracellular growth, and digestional resistance) [3,4,5]. Recent studies have reported diverse bacterial species and strains are resistant to digestion by protistan predators [6,7,8]. For example, many digestion-resistible bacteria (DRB) were found to be affiliated with alpha- and gammaproteobacteria in a number of marine ciliate species, and the bacterial secretion systems were assumed to be involved in the anti-digestion mechanism [8]. Grazing on DRB markedly affects the growth rate [9], metabolic activities [10], encystment and cyst viability [11], and the population maintenance of protistan predators [12]. So far, however, the molecular mechanisms of these bacteria-protist interactions have not been well understood, and the physiological response of protists to bacterial preys of different digestibility remains largely unknown.

Transcriptomic analysis has increasingly been exploited as a high throughput approach to understand the physiological and molecular responses of microbial eukaryotes to environmental factors and to identify the genes that are associated with specific nutritional strategies [13]. Using a transcriptomic approach, some phototrophic or mixotrophic algae (e.g., diatoms and dinoflagellates) have been studied, demonstrating specific genes involving biochemical synthesis, fatty acid oxidation, TCA cycle, nitrogen and/or iron uptakes were up- and downregulated in response to the presence of bacterial preys [14,15,16,17]. Knowledge of the metabolic processes involved in heterotrophic nutrition has been gleaned mostly from a few transcriptomic studies focused on identifying the genes involved in phagocytosis, e.g., in prey recognition, transcription regulation, and phagosomal membrane formation, as revealed in the slime mold *Dictyostelium discoideum* [18]. Nevertheless, the nutrition of free-living heterotrophic protozoa has seldom been addressed in genetic studies [13], despite that variations in gene expression pattern in protozoan species could be a window to genetically infer predator–prey interaction and ecological efficiency in the microbial loop.

*Tetrahymena thermophila* is bacterivorous but can also grow well in bacteria-free artificial medium, making it one of the best protistan models in molecular biology researches. Genome and transcriptomic information of *T. thermophila* have been reported and are well annotated in previous studies [19,20]. In this study, we took *T. thermophila* as a model for heterotrophic protozoa and hypothesized that it would have strong gene expressional responses to altered nutritional sources and to bacterial preys of contrasting digestibility. Pairwise comparisons of transcriptomes were made to identify genes that were differentially expressed between culture treatments with axenic medium, DRB, and digestible prey. This is the first study of the transcriptomic response of bacterivorous protozoa to bacterial preys with different digestibility.

## 2. Materials and Methods

### 2.1. Organisms and Cultures

The ciliate *Tetrahymena thermophila* strain CU428 was kindly provided by Prof. Shan Gao, Ocean University of China, Qingdao, China. Cells were cultured in a sterile Super Proteose Peptone (SPP) medium, which contained 1% protease peptone (Aobox Biotechnology, Beijing, China), 0.2% glucose (Solarbio, Beijing, China), 0.1% yeast extract (Oxoid, Hampshire, England), and 0.003% edetic acid (Na-Fe-EDTA, sequestrene) (Solarbio, Beijing, China).

### 2.2. Isolation and Identification of DRB Candidate Strains and Feeding Experiments

Bacterial strains of a potential digestion-resistant property were isolated and cultivated to feed *T. thermophila*. Isolation and identification of candidate DRB were carried out according to [8]. In brief, five ciliate species (*Deviata bacilliformis*, *Metopus* sp., *Paramphisiella caudata*, *Phacodinium metchnicoffi*, and *Rimaleptus mucronatus*) were isolated from soil samples and taken as protistan hosts for the DRB candidates. The ciliate cells were washed three to five times in sterilized double-stilled water and starved for 2–3 d. The cells were broken to release DRB by dropping from a height 0.5–1 m into a sterile LB medium. The DRB candidates were cultivated and isolated colonies were purified by streaking twice on agar. Identification of the bacterial strains were made by PCR amplification and sequencing of the 16S rRNA gene as previously described [8]. Taxonomic classification of these bacteria was executed using the RDP classifier pipeline [21].

**Figure 1:**
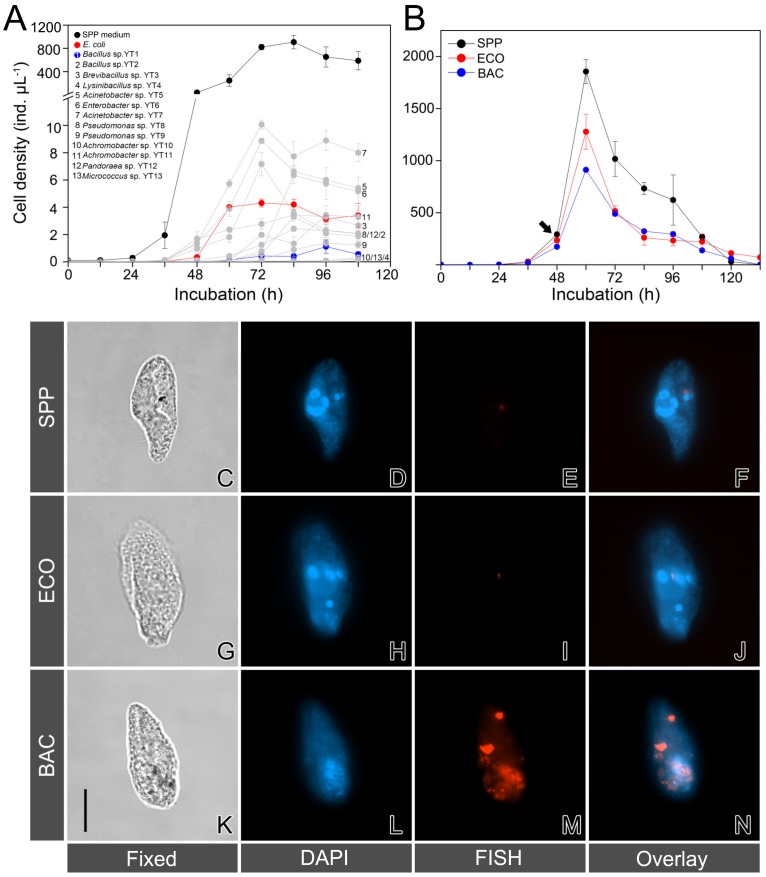
(**A**) Growth curves of *Tetrahymena thermophila* fed with water suspensions containing the selected bacterial strains (YT1–YT13), *E. coli*, and in the Super Proteose Peptone (SPP) medium. (**B**) A comparison of the growth performance of *T. thermophila* in three treatments: the axenic SPP, the SPP mixed with *E. coli* (ECO), and the SPP mixed with *Bacillus* sp. YT1 (BAC). The arrow indicates the timing point that cells were harvested for molecular analysis. Error bars represent the mean and standard errors. (**C**–**N**) Microphotographs of fixed cells of *T. thermophila* in bright field (**C**,**G**,**K**), after 4′,6-diamidino-2-phenylindole (DAPI) staining (**D**,**H**,**L**), fluoresence in situ hybridization using Cy3-labeled eubacterial probes (**E**,**I**,**M**), and overlay of DAPI and fluorescence in situ hybridization (FISH) (**F**,**J**,**N**), showing that there are no bacterial signals in the cells of the protist in the SPP treatment (**C**–**F**), few *E. coli* cells remained in the ECO treatment (**G**–**J**), and abundance of *Bacillus* sp. YT1 in the BAC treatment. (**I**–**L**). Scale bar: 20 μm.

Thirteen strains of the newly obtained bacterial strains, together with a commercial strain of *E. coli* (Trans5α, TransGen Biotech, Beijing, China), were subjected to a feeding experiment of *T. thermophila.* After incubation in the LB medium for 12 h at 30 °C with agitation of 120 r.p.m, aliquots (1 mL) of these bacterial cultures were centrifuged at 10,000× *g* for 2 min, and the pellet was resuspended in 20 mL sterile Milli-Q water. These bacterial suspensions were then used for culturing *T. thermophila*. Another culture of the ciliate with equal volume (20 mL) of SPP medium was also made as a reference. About 450 cells of *Tetrahymena* were transferred into the bacterial suspensions or medium, which were maintained in a 50-mL tissue-culture flask at 30 °C for 5 days. The abundance of ciliate cells was monitored every 12 h during the growth courses by sacrificing 20 μL of the culture solution (Figure 1A). For each culture, triplicates were set up.

Based on the performance of *Tetrahymena*, *E. coli* and a strain of *Bacillus* sp. (YT1), which supported moderate and least growth rates of the predator respectively, were selected as representatives of digestible and digestion-resistant bacteria for a second feeding experiment (Figure 1B) and for molecular preparations. Three types of medium treatments were set up: *Bacillus* sp. strain YT1 + SPP, *E. coli* + SPP, and the bacteria-free SPP medium, hereafter referred to as BAC, ECO, and SPP treatments, respectively. The two bacterial species *Bacillus* sp. and *E. coli* were cultured in LB medium at 37 °C and 120 r.p.m for 48 h. Optical densities (OD) of *Bacillus* sp. (0.740) and *E. coli* (0.904) were estimated by determining the optical density at a wavelength of 600 nm using an ultraviolet-visible spectrophotometer (model 752N, Precision and Scientific Instrument, Shanghai, China). Aliquots of 5 mL bacterial suspensions were centrifuged at 10,000× *g* for 2 min to discard the supernatants; the pellets of bacterial cells were then resuspended in 20 mL SPP medium, which were subsequently used as the medium of BAC and ECO treatments. To each of these media, 200 μL solution of antibiotics (final concentrations of penicillin: 100 units ml^−1^ and streptomycin: 100 μg ml^−1^; 100× Gibco, Life Technologies, NY, USA) was added to minimalize contamination and to inhibit bacterial growth. The effects of antibiotics on the ciliate were supposed to be offset by comparisons of these treatments. About 15 individuals of *T. thermophila* at mid-log growth stage were inoculated into the medium and incubated at 30 °C for 5.5 days, with agitation of 80 r.p.m. Each of these treatments had three replicates.

### 2.3. Fluorescence In Situ Hybridization (FISH) Assays

FISH targeting eubacterial 16S rRNA was performed to examine the presence of bacterial preys in *T. thermophila*, aiming to further verify the distinct digestibility of *Bacillus* sp. and *E. coil.* The ciliate cells were pre-cultured in axenic SPP medium or SPP medium amended with either bacterial strain. About 10 ciliate cells in mid-logarithmic phase of growth were picked out and were washed more than 3 times using sterilized Milli-Q water. The cells were then maintained in sterile water for 3 days to thoroughly digest the ingested food. The starving cells were fixed with Bouin’s solution (final concentration, 50%) on SuperFrost Plus microscopic slides (Matsunami, Osaka, Japan), which were air-dried at room temperature and stored at 4 °C in a dark place.

The FISH assay was performed as described in previous studies [22,23]. Briefly, the slides with fixed ciliate cells were rinsed 3 times in distilled water (each for 10 min), then progressively dehydrated for 5 min in each of the gradient ethanol solution (30%, 50%, 80%, and 100%). The specimens were hybridized at 46 °C for 3 h with a hybridization solution containing 0.9 M NaCl, 20 mM Tris-HCl (pH = 8.0), 0.01% SDS, 30% formamide and eubacterial universal probes (Cy3-EUB338, II and III, final concentration of 5 ng μL^−1^). After incubation, the slides were washed at 48 °C for 15 min in washing buffer (0.11 M NaCl, 20 mM Tris-HCl, 5 mM EDTA, 0.01% SDS), followed by 3 rinses with chilled Milli-Q water (each for 10 s). Finally, a mixture solution with anti-bleaching solution (Beyotime, China) and 4′,6-diamidino-2-phenylindole (DAPI) (50 ng ml^−1^) (Solarbio, Beijing, China) was dropped onto the slides. Microscopic observation was performed under an epifluorescence microscope (Olympus BX61, Tokyo, Japan) equipped with green light excitation. Photographs were captured with a Spot RT digital camera (Diagnostic Instruments, Sterling Heights, MI, USA).

### 2.4. RNA Extraction, cDNA Library Construction and Transcriptome Sequencing

The ciliate cells in the mid-log phase were sampled for RNA-Seq. Aliquots of culture medium (15 mL) containing approximately 3 × 10^6^ cells were harvested by centrifuging at 350 *g* for 5 min. The cell pellet of 150 μL was re-suspended in 750 μL RNA Protect Cell Reagent (Qiagen, Hilden, Germany) and transferred immediately to liquid nitrogen to stabilize profiles of gene expression. The total RNA was extracted with a RNeasy Plus Mini Kit (Qiagen, Hilden, Germany) according to the manufacturer’s instruction. The quality and quantity of RNA were assessed both with a NanoDrop 2000C spectrophotometer (Thermo, Wilmington, DE, USA) and by electrophoresis on a 1% agarose gel. The cDNA libraries were constructed using NEBNext^®^ Ultra^TM^ RNA Library Prep Kit (Illumina, NEB, USA). Sequencing was performed on the Illumina Hiseq 2500 platform which generated 150-bp paired-end reads. The RNA processing and cDNA sequencing were executed by a company (Novogene, Beijing, China).

### 2.5. Processing and Analysis of Transcriptomic Data

The raw reads were processed to trim the adapter sequences and filter low-quality bases (QV < 20) and short reads (<50 bp) using Trimmomatic (v0.38) [24]. The genomic data of *Tetrahymena thermophila* SB210 [19] were used as the reference and indexed using SAMtools [25] and Bowtie2 [26]. After quality filtration, the sequences of each sample were aligned/mapped against the reference genome using the TopHat (v2.1.0) with default settings [27]. The resulting Binary Alignment/Map (BAM) alignments were then subjected to Cufflinks v2.2.1 [27] to generate a transcriptome assembly. All 9 transcriptome assemblies (from 9 samples) were then merged using the *Cuffmerge* utility in the Cufflinks package. This merged assembly provided a uniform basis for further annotation and differential expression analysis. FPKM (fragments per kilobase of transcript per million fragments mapped) values of genes in each treatment were estimated using Cuffdiff v2.2.1 with default parameters [27]. To avoid false-positive estimation, only transcripts with FPKM values ≥ 1 in all the three treatments were subjected to further analysis. Differential gene expression profiles of treatment pairs (i.e., BAC vs. SPP, BAC vs. ECO, and ECO vs. SPP) were generated using Cuffdiff v2.2.1. In each pairwise comparison, a threshold (|log_2_ (fold change)| ≥ 2, and *p* ≤ 0.05) was set to define the differentially expressed genes (DEGs). The shared and unique DEGs between treatment pairs were visualized using the *VennDiagram* package in R v3.5.1 [28].

Functional annotation of the DEGs was conducted using software integrated in the DAVID web service [29]. The gene ontology (GO) enrichment of upregulated and downregulated DEGs was performed using ClueGO (v2.5.4) [30], a plug-in APP in Cytoscape [31]. In the GO enrichment analysis, Benjamini-Hochberg correction for multiple testing was used to control the *p*-values and GO term fusion option was applied to reduce the complexity of GO terms. The KEGG Ortholog (KO) assignments and pathway maps were obtained using the bi-directional best hit method (BBH) on the KEGG Automatic Ontology Annotation Server (KAAS) [32].

Identification of genes involved in lysosome, major carbon metabolism, and glutathione metabolism was conducted by the combination of KEGG and GO annotations. The DEGs involved in the following KEGG orthologies or functional annotations were extracted: lysosome (KO04142), major carbon metabolism (include glycolysis/gluconeogenesis: KO00010; TCA: KO00020; glyoxylate and dicarboxylate metabolism: KO00630), and glutathione metabolism (KO00480). To infer comprehensive expression patterns of these pathways, we also considered the significantly differentiated transcripts (SDTs) between treatments (*p* < 0.05) but with relatively lower fold changes (i.e., 0 < |log_2_ (fold change)| < 2) than we defined for the DEGs.

Principal component analysis (PCA) and multidimensional scaling (MDS) were conducted using *CummeRbund* packages in R. Several other packages, i.e., ggplot2 and LSD were also executed to visualize the DEGs with different objectives. The raw RNA-Seq data obtained in this study have been deposited in NCBI Gene Expression Omnibus (SRR10596303 to SRR10596311). The 16S ribosomal sequences of 13 bacterial strains isolated from ciliate species have been submitted to the NCBI GenBank database with accession numbers MN911365 to MN911377.

## 3. Results

### 3.1. Growth Performance of T. thermophila Feeding on Digestion-Resistant Bacteria

A total of 13 strains (YT1 to YT13) of candidate digestion-resistant bacteria were obtained from five soil ciliate species (Table S1). The RDP classification based on 16S rRNA genes showed that these strains were affiliated with four phyla and nine families: Firmicutes (Bacillaceae, Paenibacillaceae, and Planococcaceae), Gammaproteobacteria (Moraxellaceae, Enterobacteriaceae, and Pseudomonadaceae), Betaproteobacteria (Alcaligenaceae and Burkholderiaceae) and Actinobacteria (Micrococcaceae).

Not unexpectedly, *T. thermophila* grew much faster in the SPP medium than in all other suspensions containing the selected candidate DRB (Figure 1A). Among the bacterial preys, an acinetobacterial strain YT7 closely related to *Acinetobacter calcoaceticus* supported the highest growth rate (3.5 ± 0.04 d^−1^) of the ciliate, while the lowest growth rate (1.49 ± 0.20 d^−1^) appeared in the suspension of *Bacillus* sp. YT1. *T. thermophila* grew moderately in the suspension of *E. coli*, with a growth rate of (2.71 ± 0.05 d^−1^) (Figure 1A). The digestible *E. coli* strain and the digestion-resistant strain of *Bacillus* sp. YT1 were thus selected for subsequent experiments.

The growth of *T. thermophila* in the SPP medium added with *E. coli* and *Bacillus* sp. YT1 (i.e., the ECO and BAC treatments) exhibited consistent patterns (Figure 1B). The ciliate populations grew slower in both ECO (2.97 ± 0.08 d^−1^) and BAC (2.81 ± 0.07 d^−1^) than in SPP (3.16 ± 0.10 d^−1^). The difference in growth rate between SPP and BAC was statistically significant (*t*-test, *p* = 0.04); however, the other two comparisons (SPP vs. ECO and ECO vs. BAC) were not statistically significant (*t*-test, *p* > 0.05) during the early exponential phase (0–60 h) (Figure 1B).

### 3.2. Validation of Digestion-Resistant Bacteria Using FISH

The FISH assays with eubacterial probes were performed to examine whether the bacterial preys were detectable in the starving cells of *T. thermophila*. No positive signals could be detected inside the ciliate cells from the ECO and SPP treatments (Figure 1C–J). In contrast, there were consistently positive fluorescence signals of aggregated or granular bacteria within the cytoplasm of the predators in the BAC treatment (Figure 1K–N), confirming the digestibility of *E. coli* and the digestion resistance of *Bacillus* sp. YT1 for *T. thermophila.*

### 3.3. Transcriptome Overview

**Figure 2:**
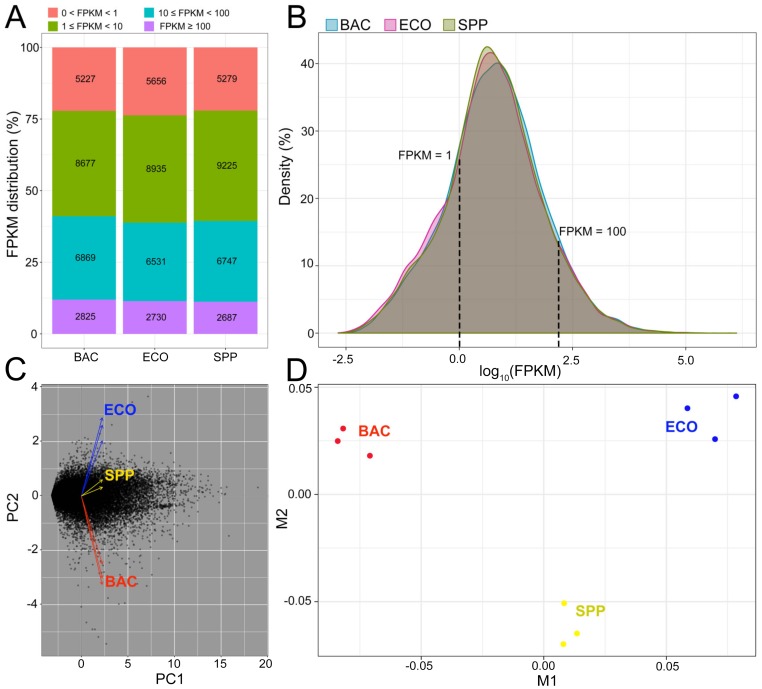
Overview of transcriptomes of *T. thermophila* cultured in the BAC, ECO, and SPP treatments, respectively. (**A**) Distribution of gene expression level in each treatment. (**B**) Density plot of the densities of the log_10_FPKM values across all genes, showing that fragments per kilobase of transcript per million fragments mapped (FPKM) ranges of the SPP (control) and two bacterial treatments were very similar, indicating no bias in the sequencing coverage among the treatments. (**C**) Principle component analysis and (**D**) multidimensional scaling based on all transcriptomic data, demonstrating the distinct gene expression pattern of the ciliate cultivated in the BAC (in red), compared to the SPP (in yellow) and the ECO treatment (in blue).

A total of 193 million paired-end (PE) raw reads were generated from nine RNA-seq libraries (three replicates for each of the three treatments). After quality control, over 186 million clean reads were retained, with the read number per library ranging from 17.2 to 24.1 million (Table S2). About 77.4%–80.9% of the reads matched unique genomic locations, while the remainders had either multiple matches (0.09%–0.12%) or were unaligned (0.06%–0.09%), which were both deprecated in the downstream analysis. A total of 24,799 genes were mapped to the reference genome of *Tetrahymena thermophila* SB210, accounting for 92.2% of the annotated genes (26,906) in the reference genome. The proportions of mapped genes were similar among three treatments, ranging from 87.7% to 89.0% (Table S2). FPKM values of the mapped genes mostly (>64%) ranged from 1 to 100 in each treatment, showing similar distributional patterns among three treatments (Figure 2A,B), which indicates that bias was limited in sequencing coverage between treatments. The transcripts (19,831 genes) with FPKM ≥ 1 were subjected to differential gene expression analysis. PCA plot of these transcripts suggested that the gene expression patterns of *T. thermophila* were highly similar within treatment, but distinct among treatments (Figure 2C). Similarly, the MDS displayed that triplicates within each treatment clustered together but were separable between treatments (Figure 2D).

### 3.4. Overview of Differentially Expressed Genes (DEGs)

**Figure 3:**
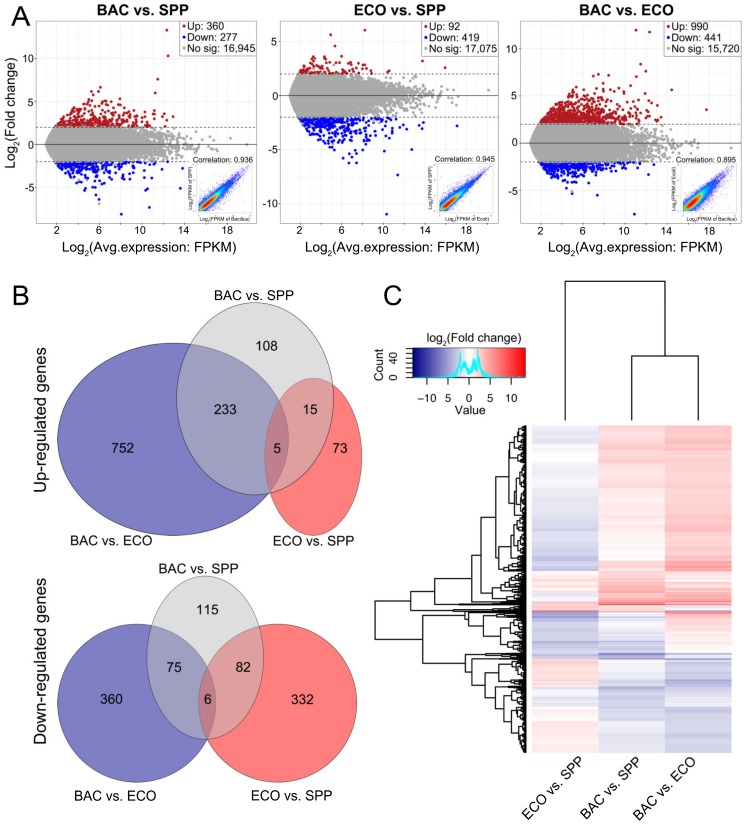
Visualization of gene expression changes of *T. thermophila* between any two of the treatments. (**A**) MA plots for the average expression and fold changes in log scale to visualize the change in gene expression and distribution of comparisons among three treatments for all 19,831 transcripts. The colored data points indicate the upregulated (in red), downregulated (in blue) and unaltered transcripts (in grey), respectively; scatter plots for each comparison of FPKM for all transcripts (inset). (**B**) Three-way Venn diagrams display the number of up- and downregulated genes that are shared and unique between these treatments. (**C**) Hierarchical cluster analysis performed on the profiles of 1919 differentially expressed transcripts, and the heatmap of log_2_(fold change) showed a distinct expression pattern in ECO vs. SPP relative to the other two comparisons.

The distribution of upregulated and downregulated DEGs was displayed by MA plots [log ratios (M) versus arithmetic mean of expression values (A)] and scatter plots (Figure 3A). In each comparison, expression level of the main component genes was linearly related between two treatment pairs (R^2^ = 0.90 to 0.95, insets in Figure 3A). A total of 1919 DEGs were identified from these comparisons, accounting for 2.9%–8.3% of the executed genes in each comparison. Compared with that in the SPP, the ciliates in the ECO treatment exhibited fewest DEGs, in which the number of the downregulated was significantly higher than those upregulated (419 vs. 92) (Figure 3A). The other two pairs comparisons resulted in more DEGs, with upregulated and downregulated DEG numbers of 360/277 (in BAC vs. SPP) and 990/441 (in BAC vs. ECO), respectively (Figure 3A).

Among the 1919 DEGs, 1185 were upregulated and 969 were downregulated among the three comparisons (some cases were repeatedly counted since they were upregulated in one comparison but downregulated in another). Only a small proportion of upregulated DEGs (20) were shared by the two bacterial treatments against SPP. Adding the DRB to SPP medium uniquely induced 341 (108 + 233) upregulated genes, which was much more than that caused by adding *E. coli* (73) (Figure 3B). The unique upregulated genes in the BAC relative to the ECO was about 10 times of that in the ECO vs. SPP (985/88), and about six times of that BAC vs. SPP (752/123). Adding *E. coli* to the axenic medium solely induced 34% of the downregulated genes (332 in 970 genes). The downregulated genes were fewer in BAC vs. SPP (190) than in ECO vs. SPP (332). In the BAC–ECO comparison, more genes were being suppressed relative to BAC–SPP (360/197) and ECO–SPP (435/414) (Figure 3B).

Distribution of the 1919 DEGs among the three comparisons was also profiled by hierarchical cluster analysis (Figure 3C). The comparisons of BAC vs. SPP and BAC vs. ECO displayed similar expressional profiles by sharing the main component of the upregulated and downregulated DEGs. However, opposite patterns occurred in the ECO vs. SPP treatments.

### 3.5. Functional Annotation and Enrichment of DEGs

**Figure 4:**
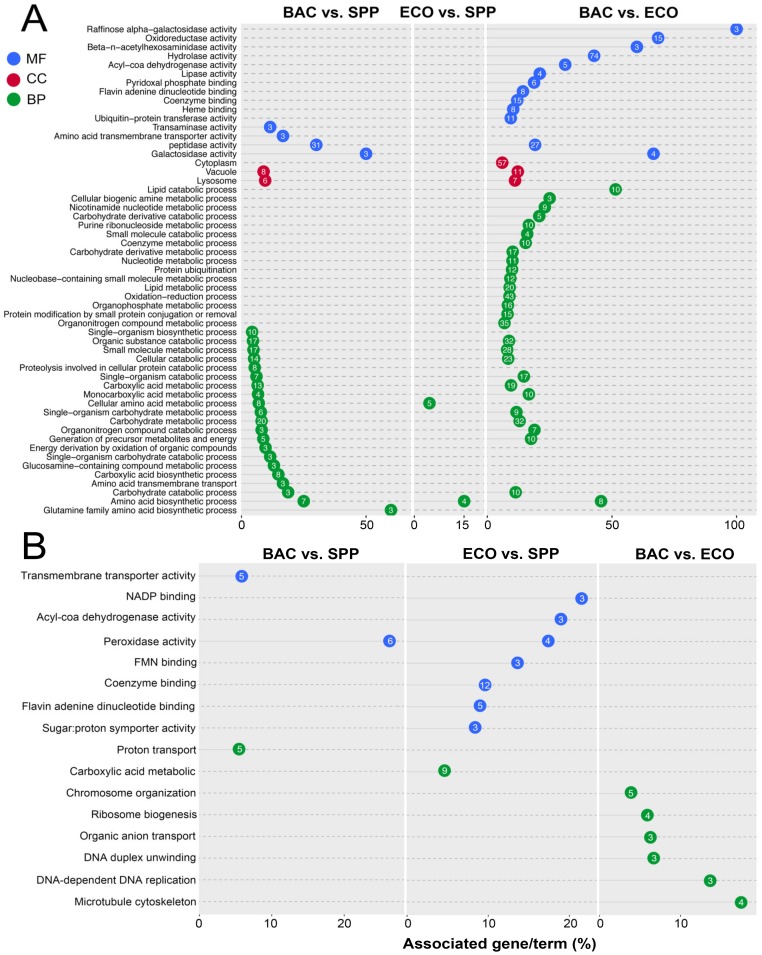
Gene ontology (GO) enrichment analysis of differentially expressed genes in *T. thermophila*. (**A**) The significantly upregulated genes (FPKM ≥ 1, log_2_ (fold change) ≥ −2, and *p* < 0.05); and (**B**) the downregulated (FPKM ≥ 1, log_2_ (fold change) ≤ −2, and *p* < 0.05) genes associated with biological process (BP), cellular component (CC), and molecular function (MF). The numbers inside the dots indicate the number of the associated genes.

There were 224 upregulated DEGs being assigned to three major functional GO terms: biological process (BP), molecular function (MF), and cellular component (CC) (Figure 4A; Tables S3 and S4). A majority of these (222 genes) were derived from the comparison pairs of BAC vs. SPP (61 genes) and BAC vs. ECO (192 genes) (Figure 4A). Forty upregulated DEGs of the BAC–SPP comparison belonged to the MF category, of which 31 were assigned to peptidase. In the BAC vs. ECO comparison, there were 114 upregulated DEGs of the MF category, with the most abundant GO terms of hydrolase activity (GO: 0016788, 0016798 and 0004553), peptidase (GO: 0008234 and 0004197), oxidoreductase activity (GO: 0016671, 016614 and 0016616) and coenzyme binding (GO: 0050662).

In the BP category, 48 upregulated DEGs were enriched to 21 GO terms in the comparison pair of BAC vs. SPP, of which 44 DEGs were of carbohydrate metabolic (GO: 0005975), organic substance catabolic (GO: 1901575), and carboxylic acid metabolic processes (GO: 0019752). Compared with the digestible bacterial treatment, upregulated DEGs (135 genes) were enriched in more GO terms (28) of BP category in the BAC treatment, of which the DEGs were mostly involved in oxidation-reductions (GO: 0055114), organic substance catabolic (GO: 1901575), organic substance catabolic (GO: 1901575), and carbohydrate metabolic (GO: 0005975) (Figure 4A).

There were eight upregulated DEGs of the cellular component category in the BAC vs. SPP comparison, which were all targeted vacuole (GO: 0005773) or lysosome (GO: 0005764). Nevertheless, 57 upregulated genes were found to be associated with cytoplasm locations (GO: 0005737) in the BAC vs. ECO comparison (Figure 4A).

Forty-four out of 969 downregulated DEGs were assigned against GO databases. The downregulated genes were enriched in 16 GO terms, which was much lower than that of the upregulated DEGs (65 GO terms). The treatment pair of ECO-SPP showed 21 downregulated DEGs, of which one third were associated with coenzyme binding (12; GO: 0050662), flavin adenine dinucleotide binding (5; GO: 0050660), and carboxylic acid metabolic (9; GO: 0019752). Meanwhile, adding *E. coli* to the medium decreased the gene expression levels of peroxidase functions (GO: 0004601). Relative to the ECO, addition of the DRB induced predominant downregulation of genes associated with cell proliferation, e.g., ribosome biogenesis (GO: 0042254), microtubule cytoskeleton (GO: 0000226), and DNA replication (GO: 0006261) (Figure 4B).

### 3.6. Transcriptional Expression Patterns Inferred from KEGG Annotations

**Figure 5:**
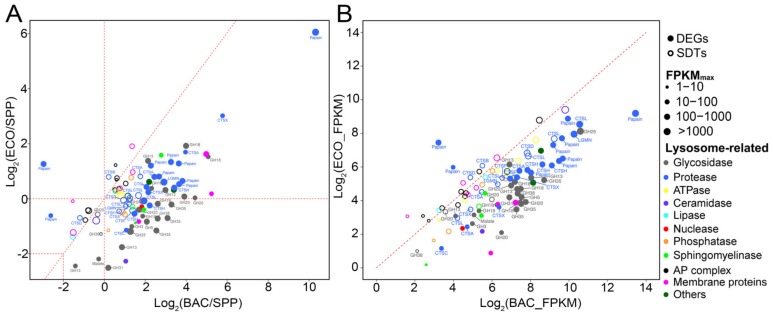
Relative expression levels of differentially expressed genes (DEGs, solid circles; |log_2_ (fold change)| ≥ 2) and significantly differentiated transcripts with relatively lower fold changes (SDTs, open circles; 0 < |log_2_ (fold change)| < 2) of *T. thermophila* associated with lysosomes in the pairwise comparisons between two bacterial treatment (BAC and ECO) and SPP (**A**), and BAC vs. ECO (**B**). Each dot represents an individual gene, and sizes of dots indicate expression abundance. Abbreviations: CTS, cathepsin; LGMN, legumain; Papain, papain family cysteine protease; GH, glycoside hydrolase.

**Figure 6:**
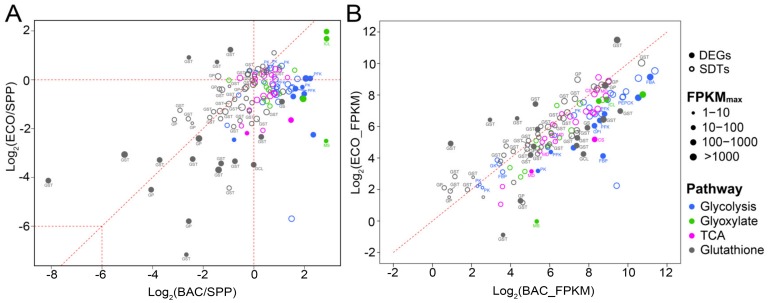
Relative expression levels of differentially expressed genes (DEGs, solid circles; |log_2_ (fold change)| ≥ 2) and significantly differentiated transcripts with relatively lower fold changes (SDTs, open circles; 0 < |log_2_ (fold change)| < 2) of *T. thermophila* involved in three carbon metabolism (glycolysis, TCA, and glyoxylate) and glutathione metabolism in the pairwise comparisons between two bacterial treatment (BAC and ECO) and SPP (**A**), and BAC vs. ECO (**B**). Each circle represents an individual gene, and its size indicates relative expression abundance. Abbreviations: GK, glucokinase; GPI, glucose-6-phosphate isomerase; PFK, 6-phosphofructokinase; FBP, fructose-1,6-bisphosphatase; FBA, fructose-bisphosphate aldolase; PK, pyruvate kinase; PEPCK, phosphoenolpyruvate carboxykinase; CS, citrate synthase; MD, malate dehydrogenase; ICL, isocitrate lyase; MS, malate synthase; GCL, glutamate-cysteine ligase; GS, glutathione synthase; GST, glutathione S-transferase; GP, glutathione peroxidase.

A total of 402 DEGs were successfully mapped to KEGG pathways (Table S5). It was found that these DEGs were most frequently involved in the pathways related to lysosome (8.5%), carbon metabolism (3.7%), and glutathione metabolism (4.2%) (Figure 5 and Figure 6). Apart from the DEGs, there were 141 significantly differentially expressed transcripts (SDTs) but with lower fold changes (0 < |log_2_ (fold change)| < 2) annotated to lysosome, carbon, and glutathione metabolisms. To provide an overview of the transcriptional patterns of individual processes involved in these pathways, we considered both the genes with high (i.e., DEGs) and with low fold changes (i.e., SDTs) (Figure 5 and Figure 6).

#### 3.6.1. Differential Expression of Lysosomal Genes

Fifty-one DEGs were mapped onto lysosome-related pathways, with 20 and 21 genes encoding proteases and glycosidases (i.e., glycoside hydrolase, GH) being the most abundant (Figure 5, Table S6). In addition, 43 lysosome-related SDTs (11 proteases and 3 glycosidases) were identified. Consistently, most DEGs and SDTs encoding proteases were upregulated in both bacterial treatments relative to these in the SPP (Figure 5A), and also highly expressed in the BAC (vs. ECO) (Figure 5B). The protease genes encoding papain-like cysteine proteinases were the most abundant (24 out of 31 genes) in the DEGs and SDTs, with a highly increased expression level of ~59 folds in the BAC, and a moderate increase by ~3.5 folds in the ECO compared to the control (Figure 5A). The papain-like DEGs and SDTs were annotated to endopeptidases cathepsin H, B, L, and exopeptidases cathepsin X and C, among which Cathepsin H has been shown as having amidase activity [33], and cathepsins B and L generally target specific peptide linkages [34,35]. Cathepsins X and C usually act as carboxymonopeptidase or carboxydipeptidase, which hydrolyze peptide bonds by removing C-terminal D- or L-amino acids [34,36].

Apart from the papain-like genes, there were seven DEGs/SDTs annotated as peptidase, among which three genes were associated with serine carboxypeptidases (cathepsin A) and two with legumains (LGMNs). These peptidase-like genes were upregulated with an average fold change of ~4.8 and ~0.62 in the BAC–SPP and ECO–SPP comparisons, respectively. Serine carboxypeptidases have protective functions towards lysosomal glycosidases GH35 [37], while LGMNs usually process enzymes and contribute to the activation of papain-like cysteine proteinases to degrade storage proteins [38].

Compared to the SPP treatment, three DEGs of glycosidase annotated to the chitinase family GH18 were found to be common in both bacterial treatments, with much higher expression levels in the BAC (4.9–33.7 folds) than in the ECO (1.3–3.8 folds) (Figure 5A,B). Nevertheless, 12 DEGs and one SDTs of glycosidase showed inconsistent expression patterns in the two bacterial vs. the SPP treatment, i.e., upregulation in the BAC–SPP (1.1–9.7 folds) comparison, but downregulation or slightly changed (0.18–1.1 folds) in the ECO–SPP (Figure 5). Among these, the most overexpressed three DEGs in the BAC–SPP comparison (by 5.8–9.7 folds, Figure 5) were affiliated to GH35, the enzymes involved in catalyzing hydrolysis of terminal *β*-galactosyl residues from carbohydrates, galactolipids, and glycoproteins [39]. The remaining 10 DEGs were found to encode *N*-acetylglucosaminidases (GH3, GH18/class II chitinases, and GH20), lysozymes (GH25, and GH18/class I chitinases), lytic transglycosylases (GH31), *α*-mannan (GH38), glucan (GH5) and *α*-starch (GH13), which are commonly involved in the degradation of polysaccharide polymers [40,41,42]. In addition, the former three enzyme types, namely *N*-acetylglucosaminidases, lysozymes, and lytic transglycosylases, were also capable of hydrolyzing bacterial peptidoglycan by cleaving glycan strands [43].

#### 3.6.2. Differential Expression of Genes Involved in Glycolytic, TCA and Glyoxylate Cycle

A total of 15 DEGs and 58 SDTs were involved in glycolysis, tricarboxylic acid (TCA) cycle and glyoxylate cycle (Figure 6; Table S7). The DEGs/SDTs assigned to each of these three pathways exhibited consistent transcriptional patterns (Figure 6) in the ECO–SPP and BAC–SPP comparisons. Only a single DEG annotated as encoding isocitrate lyase (ICL) was highly expressed by 3.2 folds in the ECO–SPP (Figure 6A). The expression level of this gene was even higher, about 7.3 folds in the BAC–SPP comparison (Figure 6B). ICL involved in the glyoxylate cycle catalyzes the cleavage of isocitrate to succinate and glyoxylate [44].

Other DEGs/SDTs, including 11/46 DEGs/SDTs involved in glycolysis and the TCA cycle and 3/12 DEG/SDTs involved in the glyoxylate cycle showed inconsistent expression patterns in the two bacterial vs. the SPP treatments, i.e., most upregulation in the BAC–SPP (0.59–7.3 folds) comparison, but downregulation or slightly changed (0.18–3.9 folds) in the ECO–SPP (Figure 6). These DEGs/SDTs were annotated to more than one copy of the enzymes involved in each step of the glycolysis pathway and TCA cycle (Figure S2). Especially, the DEGs included two unidirectional enzymes controlling the metabolism rate, i.e., 6-phosphofructokinase (PFK) and pyruvate kinase (PK) (Figure S2). PK is also responsible for the production of net ATP and pyruvate in the glycolytic flux [45], and indirectly affects the activity of TCA cycle by the producing pyruvate. Another two notable enzymes were the ones involved in the gluconeogenesis process, i.e., fructose-1,6-bisphosphatase (FBP) and phosphoenolpyruvate carboxykinase (PEPCK) (Figure S2). Functionally, FBP is an enzyme that converts fructose-1,6-bisphosphate to fructose 6-phosphate in the gluconeogenesis process [46], while PEPCK is involved in the catabolism of carbon skeletons of amino acids [47].

#### 3.6.3. Glutathione Metabolism

The expression levels of most DEGs (12 out of 17 genes) and SDTs (26 out of 40 genes) involved in glutathione (GSH) metabolism consistently decreased in these two bacterial treatments compared to the SPP. The sharpest decreases appeared in several cases, e.g., up to 1/278 folds in one GST-like DEG and 1/55 folds in a GP-like DEG (Figure 6A; Table S8). However, the expression level of the glutathione metabolism DEGs and SDTs were not significantly different between the BAC and ECO (Figure 6B). There were 12 DEGs assigned to glutathione S-transferase (GST) and three DEGs to glutathione peroxidase (GP), of which the expression levels decreased by 1.7–278 folds in the bacterial treatments compared to SPP. The remaining two DEGs, which were associated with glutamate-cysteine ligase (GCL) and glutathione synthase (GS), were significantly suppressed in the ECO (0.09/0.53 folds), but only slightly decreased/increased in the BAC–SPP treatment (1.0/2.2 folds) (Figure 6). Functionally, GST and GP serve as important lines of defense against reactive oxygen species [48], while GCL and GS are key enzymes involved in the progress of GSH synthesis [49,50].

## 4. Discussion

By using *Tetrahymena thermophila* as a model, we were able to manipulate the culture medium and demonstrate the effects of food source on phenotypic changes of protistan grazers. We observed that *T. thermophila* grew much faster in the bacteria-free SPP medium than in the SPP medium mixed with either *E. coli* or *Bacillus* sp. YT1 (Figure 1B). It is likely that the ciliate species has adapted to the artificial, bacteria-free medium during the long history of the culture, so re-adaptation to the bacterial foods might have induced dramatic physiological shifts by increasing the energy cost and decreasing the efficiency in digestion, absorption, and cell division. The FISH assay partly supports this notion in that a number of *Bacillus* cells, but not of *E. coli* cells, were still viable inside the *Tetrahymena* cells after being ingested for a while (Figure 1C–N). Our study also showed that *T. thermophila* grew at different rates when fed with these two species of bacterial preys, and that the addition of digestion-resistant bacteria (*Bacillus* sp. YT1) into the SPP medium resulted in a much lower growth rate (Figure 1A), which was consistent with many previous studies showing that some ingested bacteria cannot be effectively digested to supply nutrients for growth of protistan grazers [8,9,51,52]. More importantly, using comparative transcriptomics we revealed for the first time the metabolic and molecular responses (particularly of digestion and energy use-related biochemical processes) that may underlie the phenotypic variations of the model protist *T. thermophila* under three feeding conditions, i.e., the bacteria-free medium (SPP), the medium with digestible bacteria (ECO), and the medium with digestion-resistant bacteria (BAC).

### 4.1. Increased Protistan Lysosomal Protease, Chitinase, and Glyoxylate Cycle-Related Activities for Digesting Bacterial Preys

Compared with the bacteria-free treatment, most of the protease DEGs in these two bacterial treatments were upregulated (Figure 4), indicating cellular responses of *T. thermophila* involving both ingestion and digestion of bacterial preys. A similar observation was reported in an enzymatic study of *T. pyriformis*, which had markedly increased acid proteinase activities when feeding on yeast cells vs. axenic medium [53]. These upregulated genes encode proteases of versatile functions, such as cleaving proteins, peptides and protein-containing compounds (e.g., papain-like cathepsins and some peptidase), or are related to enzymes with protective or active functions towards other lysosomal enzymes (e.g., cathepsin A and legumain). Among these upregulated protease DEGs, the genes encoding papain-like cysteine proteinases were found to be the most abundant (Figure 5), suggesting the formation of phagocytic vesicles during ingestion of microbial cells could have been a major trigger for the higher production of proteases in *T. thermophila*. This notion is supported by the study of a pathogenic protist *Entamoeba histolytica*, in which the cysteine proteinases were incorporated into phagocytic vesicles for intercellular digestion following phagocytosis of erythrocytes [54]. Furthermore, the higher expressional levels of lysosomal proteases might be a response to the increased quantity and diversity of substrates (bacterial proteins) in both *E. coli* and *Bacillus* treatments. The proteases with either endopeptidase or exopeptidase activity we found in the present study may function in hydrolyzing peptidoglycans [35,43], which are major components in bacterial cell walls but are apparently absent in the SPP medium. Bacterial protein composition is well known to be complicated; for example, the cytosolic proteome of *E. coli* comprises more than 1000 different proteins and around two million soluble proteinous molecules [55,56].

It was interesting to observe that bacterial additions of both *Bacillus* and *E. coli* triggered higher expression of three glycoside hydrolase genes encoding chitinases (GH18 chitinases, classes I and II) in *Tetrahymena* (Figure 5), as chitin is not rich in both bacteria species. A possible explanation is that the protistan chitinases may act as a lysozyme to participate in lysing bacterial cell walls, since it has been demonstrated that some bacterial chitinases share a conserved core in the protein structure with lysozyme [57], thus are functionally similar to the common digestive enzymes (lysozymes) in catalyzing the cleavage of *β*-1, 4-glycosidic linkage of peptidoglycans in cell walls of bacteria [58]. This also explains why there was a much higher expression level of GH18 of *Tetrahymena* during digesting peptidoglycan-rich *Bacillus* cells relative to peptidoglycan-poor *E. coli*.

Few carbon metabolism-related DEGs were commonly up- or downregulated in the DRB and digestible bacterial treatments (Figure 6), indicating there are great variations in carbon metabolisms of *T. thermophila* during digestion of different bacterial preys. The only DEG commonly upregulated in both bacterial treatments was found to encode an ICL, which facilitates the glyoxylate cycle (GC) pathway with another enzyme (malate synthase), allowing cells to consume irregular carbon sources such as acetate or fatty acids [59]. The glyoxylate cycle has been reported to allow plants and bacteria to subsist on acetyl-CoA or other two carbon compounds when the regular carbon sources (glucose and fructose) are in shortage [60,61]. In nematodes and yeasts, upregulation of ICL, or an enhancement of the glyoxylate cycle, has been induced by starvation or shortage of regular carbon sources (glucose and fructose), and when acetate and fatty acid were the only carbon nutrient [62,63]. It is thus possible that *T. thermophila* in both bacterial treatments was subject to a shortage of carbon source compared with that in the SPP medium. After all, mixing protein-rich bacterial cells with SPP increased the ratio of protein to glucose of the medium, providing fewer amounts of glucose to the protist during a unit of time.

Relative to the SPP, *Tetrahymena* fed with *E. coli* exhibited downregulation of carbon metabolism-related genes involved in glycolysis, TCA and glyoxylate cycle (MS gene), suggesting weaker glycolytic activity and less energy production during metabolizing protein-rich bacterial carbon sources in digestible bacteria treatment. This observation in the heterotrophic protist is different from previous findings for a mixotrophic alga *Ochromonas*, which had upregulated expression of the genes encoding for unidirectional enzymes GCK and PFK, implying a higher glycolytic activity when feeding on a bacteria prey [15]. A mixotrophic haptophyte *Prymnesium parvum* feeding on bacteria and ciliates also exhibited upregulation of the genes involved in TCA and the glyoxylate cycle [16]. These contradictory results for the heterotrophic *Tetrahymena* and the mixotrophs might be related to their different trophic lifestyles. The bacterial prey might have provided an extra organic carbon source in the form of fatty acids to *Prymnesium parvum*, resulting in the higher expression of glycolysis for producing energy [16], and the increased expression of carbon metabolism in *Ochromonas* might be due to extra phosphoenolpyruvate converted from bacterial amino acids by the enzyme phosphoenolpyruvate carboxykinase (PEPCK) [15]. However, neither fatty acid pathways nor PEPCK were found to be overexpressed in *Tetrahymena* feeding on *E. coli* compared to the SPP treatment; perhaps it was because the SPP medium contains more sugars than the SPP mixed with digestible bacterial cells, which might also be different from the sugar-depleted mediums used to culture those mixotrophic protists [15,16].

### 4.2. Higher Expression Levels of Protease, Glycosidase, and Carbon Metabolism-Related Genes in Response to DRB vs. Digestible Bacteria

Comparisons of transcriptomics of the BAC and ECO treatments provide clues for the mechanisms of how *Tetrahymena* specifically responded to the digestion-resistant *Bacillus*. The higher expressional levels of protease-encoding genes in BAC than in ECO treatments were probably due to several aspects: (1) the Gram-positive bacteria (e.g., *Bacillus*) have richer peptidoglycan in cell walls than Gram-negative ones (e.g., *E. coli*) [64], and more protein-degrading enzymes were therefore expressed to obtain organic matter and nutrients from *Bacillus*; (2) *Bacillus* might have formed spores during the antibiotic pretreatment or after being ingested. The coat of a spore is composed of several distinct layers and ~30 extensively cross-linking proteins [65,66]. Coats might have triggered the expression of the protein-degrading enzymes or have consumed some of these enzymes to provide a barrier against lytic enzymes that can degrade the peptidoglycan cortex lying below them [67]. Unfortunately, *Bacillus* spores could not be easily detected using conventional FISH protocols [68] and the existence of spores inside the ciliate has to be further verified.

We also identified that GH genes affiliated with *N*-acetylglucosaminidases (GH3, GH18/class II chitinases, and GH20), lysozymes (GH25, and GH18/class I chitinases), lytic transglycosylases GH35, GH13 (for *α*-starch), GH5 (for glucan), and GH38 (for *α*-mannan) in *Tetrahymena* were significantly downregulated (or non-differential) in the ECO but upregulated in the BAC treatment compared with the SPP (Figure 5). Lower or non-differential expression of glycosidases and lysozymes was also observed in the microarray study for a heterotrophic protist *Dictyostelium* feeding on *E. coli* (vs. axenic medium) [18] and in transcriptomic studies for the mixotrophic chrysophyte *Ochromonas* feeding on heat-killed bacteria [15,17]. Protein is usually richer than carbohydrates in a bacterial cell [69,70]. All these suggest that lower expression of these GH family genes in the ciliated protozoa could be an indication of trophic processes involving digestible bacteria.

**Figure 7:**
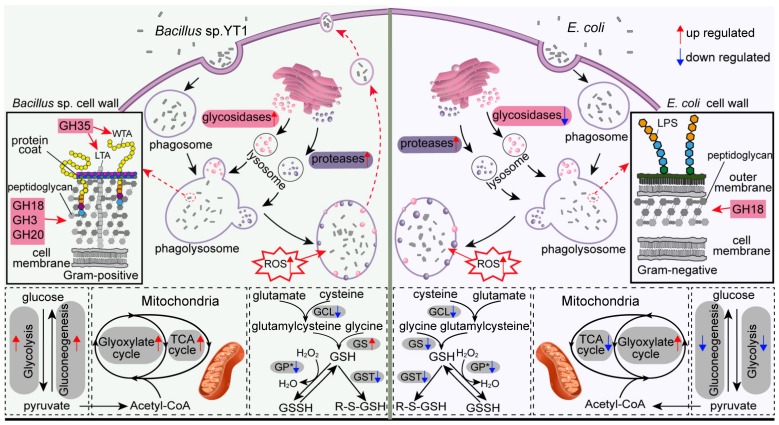
Schematic model illustrating the phagocytic processes and genes expression changes in *T. thermophila* under three treatments. Abbreviations: WTA, wall teichoic acid; LTA, lipoteichoic acid; LPS, lipopolysaccharide; ROS, Reactive oxygen species; GCL, glutamate-cysteine ligase; GS, glutathione synthase; GST, glutathione *S*-transferase; GP, glutathione peroxidase.

The upregulation of these GH genes in the BAC treatment could be, again, due to the chemical composition of cell wall of *Bacillus*. For example, the GH35 (*β*-galactosidases), which hydrolyzes the terminal *β*-galactosyl residues in carbohydrates, galactolipids, and glycoproteins [39], was among the highest upregulated in the BAC–SPP comparison (Figure 6A), implying a specific response of *Tetrahymena* in digesting teichoic components in Gram-positive bacteria since this phosphorylated polysaccharide is exclusively present in the cell walls of Gram-positive bacteria (Figure 7), including *Bacillus* [71,72,73]. Furthermore, we did not observe an accumulation of *Bacillus*-containing food vacuoles; it is thus highly likely that the undigested *Bacillus* may be egested in small vacuoles from the predator which simultaneously causes the loss of digestive enzymes, inducing higher expression and translation of GHs to compensate the digestive capability for survival (Figure 7). This potential mechanism is further supported by the enhancements of the glycolytic and TCA pathways (see below). Previous studies have showed that protists, e.g., *Acanthamoeba* spp. and *Tetrahymena* sp. were able to expel fecal pellets that contained bacterial cells into the extracellular environment [74,75].

The digestibility of bacterial preys seemingly caused significant shifts in carbon metabolic processes related to glycolysis, TCA and glyoxylate cycles, of which the expressions were consistently upregulated in the BAC vs. ECO treatments (Figure 6). These included two unidirectional enzymes, 6-phosphofructokinase (PFK) and pyruvate kinase (PK), which potentially control the metabolism rate of glycolysis or regulate the energy production through glycolysis and TCA [76]. Upregulation of both PFK and PK in *Tetrahymena* in the BAC treatment suggests a higher level of generation of energy and pyruvate during digestion of DRB (Figure 7). Moreover, the upregulation of carbon metabolism in the BAC is probably related to the highly expressed lysosomal genes in the same treatment. Apart from the common function of prey digestion, the lysosome has been proposed to be a control center for energy metabolism [77]. The generation and maintenance of the lysosomal pH gradient requires the activity of a proton-pumping v-type ATPase, which uses the energy of ATP hydrolysis to pump protons into the lysosomal lumen [77].

The gene encoding the PEPCK enzyme of *Tetrahymena* was also highly expressed in the BAC treatment than ECO (0.53 folds), implying a more active catabolism of amino acids in the glycolysis pathways during digestion of the DRB vs. the digestible *E. coli* (Figure 7). The PEPCK is involved in catabolism of the carbon skeletons of amino acids (e.g., glutamine and glutamate) in glycolysis, in which the amino acids are usually oxidized to oxaloacetate and further converted by PEPCK into phosphoenolpyruvate (PEP), which is then turned to pyruvate (via pyruvate kinase) for subsequent oxidation in the TCA as acetyl-CoA (Figure 7) [47]. Apparently, more assimilated amino acids had been diverted to energy production rather than synthesis of biomass in the slowly growing protist predating DRB.

### 4.3. Lower Expression of Glutathione (GSH) Metabolism in Tetrahymena feeding on Bacteria

In both bacterial treatments, *Tetrahymena* had marked downregulation of the genes involved in GSH-related enzymes. It has been reported that GSH promotes a reducing redox status and contributes to approximately 90% of cellular antioxidants in a cell [78,79,80], thus inducing a decrease in the phagocytic function in macrophages [81]. The sharp decrease of GSH synthesis in *T. thermophila* might allow the persistence of intracellular reactive oxygen species (ROS) and reactive nitrogen species (e.g., O_2_^−^, H_2_O_2_, ^●^OH, HOCl and NO^●^), which are well known for playing an important role in bacterial phagocytosis and killing via various mechanisms in immune cells (macrophages and polymorphonuclear leukocytes) of mammals [82]. Furthermore, GSH has also been reported to be positively correlated with growth rate [83], and to modulate cell proliferation [84,85] and DNA synthesis [86,87], which are potential explanations for the bacteria-induced decrease of GSH synthesis in *T. thermophila*.

## 5. Conclusions

Protistan ingestion and digestion of bacteria are important steps in channeling particulate organic matter to higher trophic levels via the microbial loop. These ecological processes depend on many factors including physiology of the grazers and biological property of bacterial preys. Our study is the first to investigate protistan transcriptomes in response to bacterial preys of different digestibility. We found that the expression pattern of functional genes of *T. thermophila* was distinct in different medium/bacterial treatments, which contributes to a better understanding of the biological mechanisms underlying the distinct growth efficiency of protists. These findings have implications for the modeling of status and functioning of protistan bacterivory, in which bacterial property might be parameterized as it is among major factors driving the protistan physiology, especially in ingestion and digestion processes. Phylogenetic resolution of bacterial communities may partially reflect the bacterial property of digestibility, and metatranscriptomes provide information of protistan metabolisms. Bringing these two aspects into parameterized models, we may step forward towards a better predictive capability of the microbial loop function in complexed natural systems.

## Supplementary Materials

Supplementary materials can be found at https://www.mdpi.com/2076-2607/8/4/559/s1, Figure S1: Relative expression levels of differentially expressed genes (DEGs) of T. thermophila associated with different metabolic pathways. Each dots represents an individual gene. Figure S2: A heatmap showing differential expressed genes associated with major carbon metabolism and glutathione metabolism in *T. thermophila* in the bacteria-free medium SPP, the SPP mixed with digestible *E. coli* (ECO) and with digestion-resistant bacteria (BAC). The value of pairwise comparison refers to log_2_(fold change); Table S1: The closest matches in the Genbank by BLASTing 16S rRNA genes and RDP classification of 13 strains of digestion-resistant bacteria isolated from five ciliate species; Table S2: Summary of RNA-Seq reads of *Tetrahymena thermophila* and their matches to the reference genome of *T. thermophila* SB210. Table S3: Gene ontology (GO) enrichment analysis of upregulated differentially expression genes (DEGs) in *T. thermophila*; Table S4: Gene ontology (GO) enrichment analysis of downregulated differentially expression genes (DEGs) in *T. thermophila*; Table S5: Expression levels of 402 DEGs which successfully mapped to different KEGG pathways in *T. thermophila*; Table S6: Expression levels of lysosome related genes in the form of FPKM (Fragment per kilobase exon per million fragments) with the original fragment counts in different treatments. Relative expression levels of those genes between each two treatments are also listed.; Table S7: Expression levels of genes involved in major carbon metabolisms in the form of FPKM (Fragment per kilobase exon per million fragments) with the original fragment counts in different treatments. Relative expression levels of those genes between each two treatments are also listed; Table S8: Expression levels of glutathione-related genes in the form of FPKM (Fragment per kilobase exon per million fragments) with the original fragment counts in different treatments. Relative expression levels of those genes between each two treatments are also listed.
