# Supplementary material for: Comparative Transcriptomics Reveals Distinct Gene Expressions of a Model Ciliated Protozoan Feeding on Bacteria-Free Medium, Digestible, and Digestion-Resistant Bacteria
Source: Microorganisms. 2020 Apr 13;8(4):559. doi: 10.3390/microorganisms8040559 (PMC7232342; doi:10.3390/microorganisms8040559)
Supplement: Supplementary file 1 [file microorganisms-08-00559-s001.zip › Table S1.pdf]

Table S1. The closest matches in the Genbank by BLASTing 16S rRNA genes and RDP classification of 13 strains of digestion-resistant bacteria isolated from five ciliate species.

Strains ID	Accession number	Closest matched species (accession number)	Coverage (%)	Identity (%)	Classification	Ciliate hosts
YT1	MN911365	*Bacillus* sp. 'CIFT MFB 14700 AVL8' (KP240995)	98	100	Firmicutes, Bacillaceae	*Paramphisielb caudata*
YT2	MN911366	*Bacillus thuringiensis* strain 262AG1 (KF836518)	99	99	Firmicutes, Bacillaceae	*Paramphisielb caudata*
YT3	MN911367	*Brevibacillus* sp. C402 (HQ704704)	98	99	Firmicutes, Paenibacillaceae	*Phacodinium metchnicoffi*
YT4	MN911368	*Lysinibacillus fusiformis* strain BH45 (KY910256)	98	99	Firmicutes, Planococcaceae	*Paramphisielb caudata*
YT5	MN911369	*Acinetobacter soli* strain GFJ2 (CP016896)	99	99	Gamma, Moraxellaceae	*Deviata bacilliformis*
YT6	MN911370	*Enterobacter hormaechei* strain PY12 (KC759162)	99	99	Gamma, Enterobacteriaceae	*Paramphisielb caudata*
YT7	MN911371	*Acinetobacter calcoaceticus* strain L22 (JN700142)	99	99	Gamma, Moraxellaceae	*Paramphisielb caudata*
YT8	MN911372	*Pseudomonas luteola* strain FQ17 (MF144465)	98	99	Gamma, Pseudomonadaceae	*Paramphisielb caudata*
YT9	MN911373	*Pseudomonas aeruginosa* strain RHH13 (HQ143612)	99	99	Gamma, Pseudomonadaceae	*Paramphisielb caudata*
YT10	MN911374	*Achromobacter* sp. A3 (KT316387)	98	99	Beta, Alcaligenaceae	*Metopus* sp.
YT11	MN911375	*Achromobacter xylosoxidans* strain E2 (MK849863)	98	99	Beta, Alcaligenaceae	*Deviata bacilliformis*
YT12	MN911376	*Pandoraea vervacti* strain NS15 (CP010897)	99	99	Beta, Burkholderiaceae	*Rimaleptus mucronatus*
YT13	MN911377	*Micrococcus luteus* strain HN-40 (KT003279)	99	99	Actino, Micrococcaceae	*Metopus* sp.
