# Supplementary material for: Comparative Transcriptomics Reveals Distinct Gene Expressions of a Model Ciliated Protozoan Feeding on Bacteria-Free Medium, Digestible, and Digestion-Resistant Bacteria
Source: Microorganisms. 2020 Apr 13;8(4):559. doi: 10.3390/microorganisms8040559 (PMC7232342; doi:10.3390/microorganisms8040559)
Supplement: Supplementary file 1 [file microorganisms-08-00559-s001.zip › Table S2.pdf]

Table S2. Summary of RNA-Seq reads of *Tetrahymena thermophila* and their matches to the reference genome of *T. thermophila* SB210.

**Treatment**	**Biological replicate**	**Raw reads pairs**	**Clean reads pairs**	**Mapped reads**		**Mapped pairs**		**Matched genes (%) (FPKM ≥0）**	**Transcripts (%) (FPKM ≥1）**
				**Left**	**Right**	**Unique (%)**	**Non-unique (%)**		
BAC	1	24,387,103	23,271,702	20,401,554	20,144,428	18537592 (79.7)	47000 (0.2)	23598 (87.7)	18371 (68.3)
	2	17,961,118	17,153,178	15,139,688	15,039,796	13880451 (80.9)	30278 (0.2)		
	3	20,033,948	19,322,249	16,953,688	16,513,810	15177329 (78.5)	35742 (0.2)		
ECO	1	22,200,785	21,559,289	18,941,210	18,934,625	17409636 (80.8)	34688 (0.2)	23852 (88.6)	18196 (67.6)
	2	23,322,428	22,676,113	19,493,649	19,405,437	17556749 (77.4)	40579 (0.2)		
	3	21,141,397	20,455,088	17,785,948	17,472,435	15936233 (77.9)	37276 (0.2)		
SPP	1	20,123,091	19,296,448	16,762,693	16,522,502	15091602 (78.2)	33121 (0.2)	23938 (89.0)	18659 (69.3)
	2	24,582,948	24,096,730	21,185,391	21,188,796	19451728 (80.7)	37736 (0.2)		
	3	19,706,340	19,023,151	16,333,786	16,294,713	14787354 (77.7)	34996 (0.2)		
**Total**		193,459,158	186,853,948	162,997,607	161,516,542	147,828,674	331,416	24799 (92.2)	19831 (73.7)
