# Supplementary figures and images for: Comparative Transcriptomics Reveals Distinct Gene Expressions of a Model Ciliated Protozoan Feeding on Bacteria-Free Medium, Digestible, and Digestion-Resistant Bacteria
Source: Microorganisms. 2020 Apr 13;8(4):559. doi: 10.3390/microorganisms8040559 (PMC7232342; doi:10.3390/microorganisms8040559)

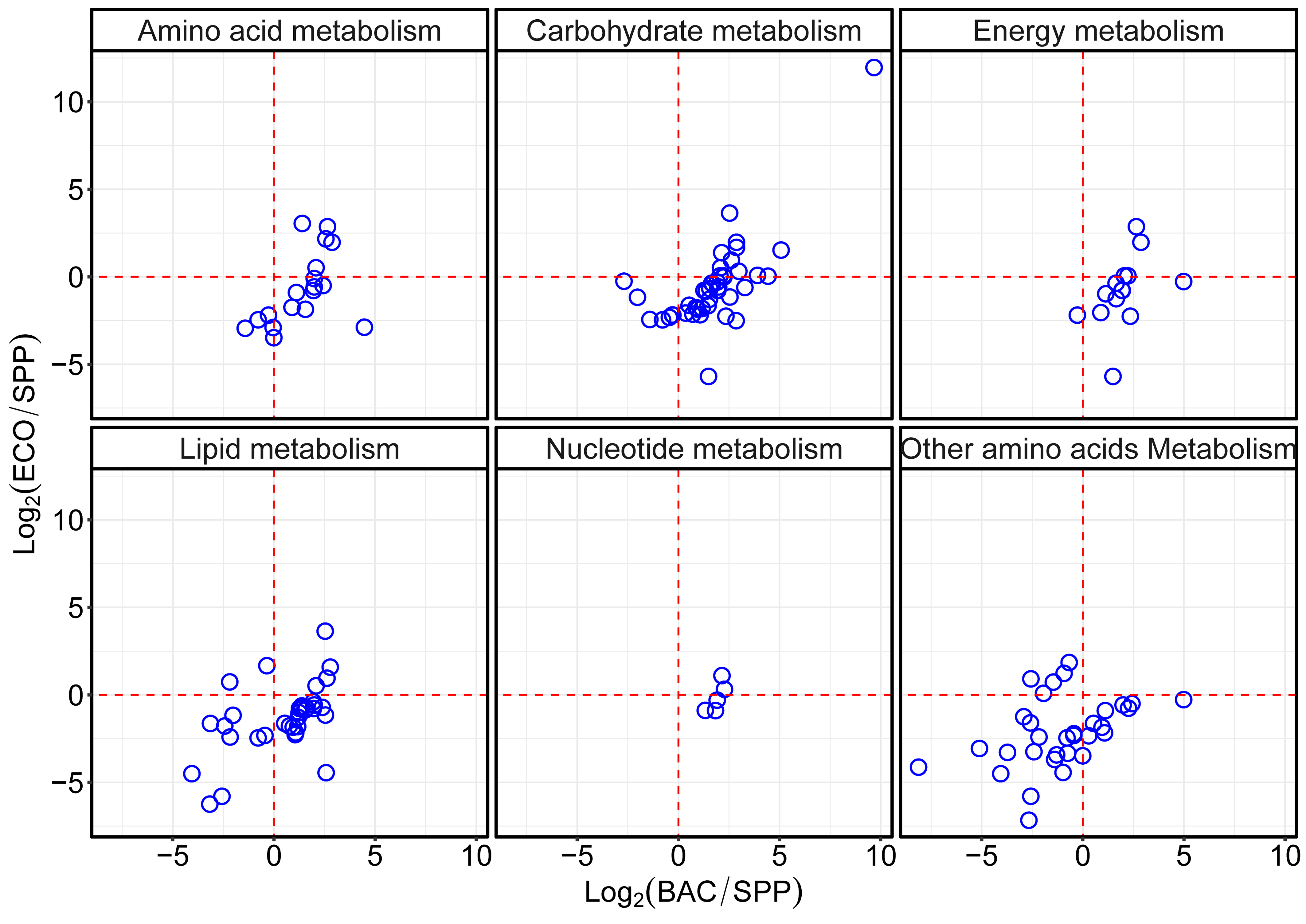

Supplement: Supplementary file 1 [file microorganisms-08-00559-s001.zip › Figure S1.tif]

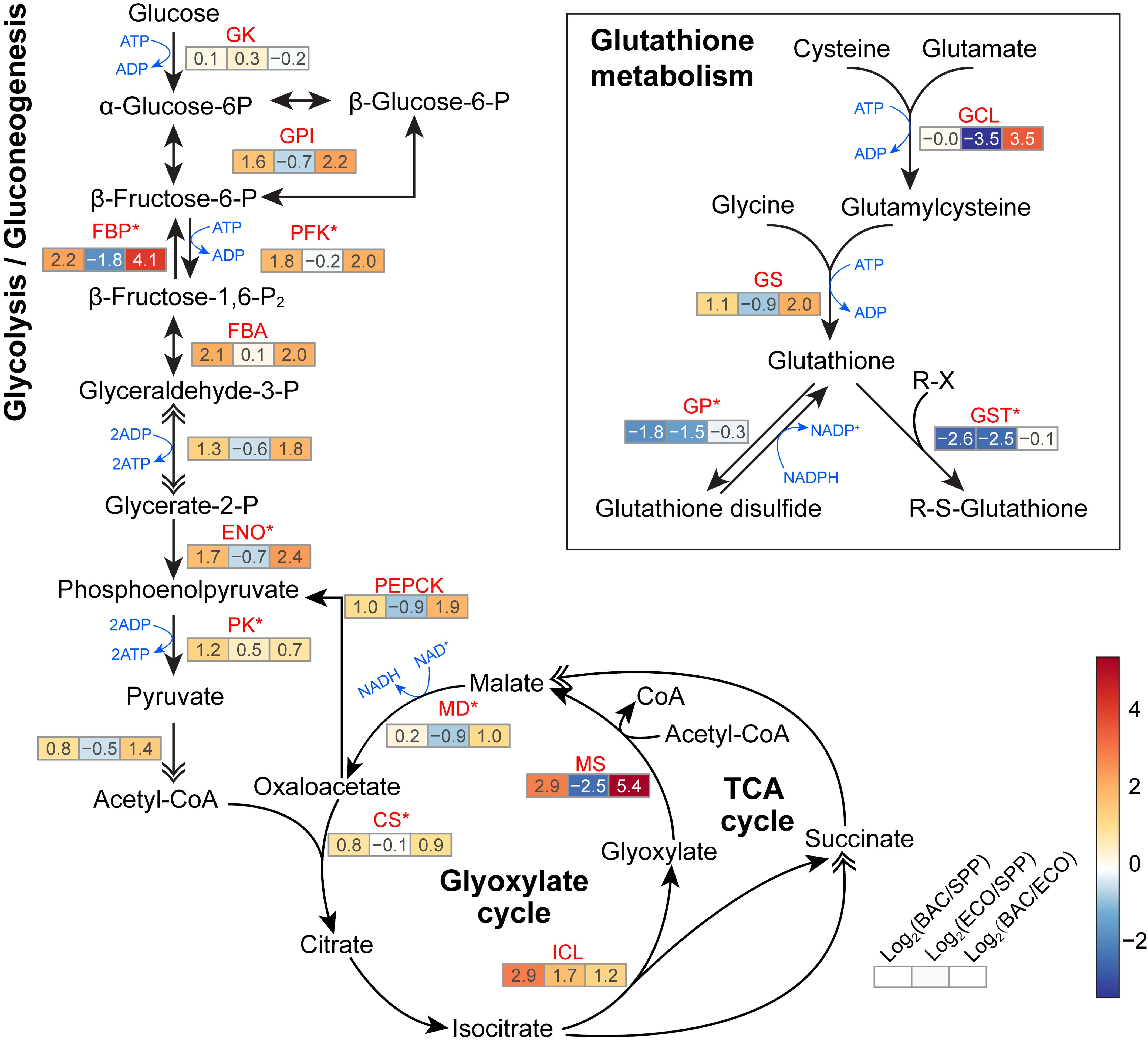

Supplement: Supplementary file 1 [file microorganisms-08-00559-s001.zip › Figure S2.tif]
